# Outpatient cardiovascular diseases and diabetes medicines dispensing in the population with government health insurance in Syria between 2018 and 2019: a retrospective analysis

**DOI:** 10.1186/s12913-021-07124-6

**Published:** 2021-10-13

**Authors:** Saleh Aljadeeah, Eckhard Nagel, Veronika J. Wirtz

**Affiliations:** 1 University of Bayreuth, Institute of Medical Management and Health Sciences, Prieserstr. 2, 95440 Bayreuth, Germany; 2 Department of Global Health, Boston University School of Public Health, 801 Massachusetts Avenue, 3rd floor, Boston, MA 02118 USA

**Keywords:** Non-communicable disease, Cardiovascular disease, Diabetes, Medicine, Insulin, Dispensing data, Demography, Syria, Conflict

## Abstract

**Background:**

Low- and middle-income countries bear the highest burden of non-communicable diseases (NCDs) mortality and morbidity. Syria has undergone an epidemiological transition from infectious diseases to NCDs in the past decades. Despite the high prevalence of cardiovascular diseases (CVDs) and diabetes in Syria, little is known about medicines utilization or prescriptions for these diseases. The aims of this study are to present the patterns and rates of dispensing medicines used for CVDs and diabetes among patients with government health insurance in Syria and examine age, sex, and regional variation in the dispensing of these medicines.

**Methods:**

Outpatient data from June 2018 to May 2019 on dispensed medicines for 81,314 adults with government health insurance were obtained. The dispensing rate was expressed as the number of defined daily doses (DDDs) per 1000 beneficiaries per day (DID). The DID is a measurement that is used in drug utilization research to control for differences or changes in population size between or within countries. The number of DIDs was adjusted according to beneficiaries’ sex, age, and governorate.

**Results:**

Beneficiaries received 302.09 DIDs of CVDs medicines and 35.66 DIDs of diabetes medicines, including 0.96 DID of insulin (2.99% of the total of diabetes medicines). CVDs and diabetes medicine dispensing rates were low during the study period and included very low rates of insulin dispensing compared to the dispensing rates of these medicines in other countries in East Mediterranean Region or in Europe. We found lower dispensing rates of CVDs medicines among female beneficiaries (249.59 DIDs) than male beneficiaries (388.80 DIDs). Similarly, the dispensing rates of diabetes medicines among female beneficiaries (29.42 DIDs) were lower than those among male beneficiaries (45.98 DIDs). In addition, there were lower rates of CVDs and diabetes medicines and very low to no dispensing of insulin in some governorates that were partly controlled by the Syrian government compared to other governorates that were completely or mostly controlled by the Syrian government.

**Conclusions:**

Additional efforts are needed to raise awareness about the prevention and management of CVDs and diabetes especially among females in Syria and consider cultural issues that might influence access to healthcare services. There is a crucial need to address the political and geographical challenges caused by the conflict which have limited access to CVDs and diabetes medicines in some regions in Syria.

**Supplementary Information:**

The online version contains supplementary material available at 10.1186/s12913-021-07124-6.

## Background

Non-communicable diseases (NCDs) account for 71% of all deaths globally. Seven NCDs were in the top 10 causes of death in 2019 where cardiovascular diseases (CVDs) and diabetes are listed among the four leading causes of death globally [1, 2]. Low- and middle-income countries (LMICs) bear the highest burden of NCDs mortality and morbidity [3]. In particular, CVDs and diabetes are underdiagnosed and undertreated in LMICs and result in considerable morbidity and mortality [4]. In 2019, 463 million people were living with diabetes worldwide, with approximately 80% of those with the disease living in LMICs [5]. Similarly, over 75% of deaths caused by CVDs in 2019 occurred in LMICs [6]. It is estimated that this disease burden will continue to rise and cause financial burdens on health systems and households, particularly in LMICs [3].

Syria has undergone an epidemiological transition from infectious diseases to NCDs in the past decades. Before the onset of the conflict in 2011, NCDs (mainly CVDs, cancer, and diabetes) accounted for 77% of total mortality in the country [7, 8], with CVDs estimated to account for 25% of all deaths in 2016 in Syria [9]. Since 1980, the prevalence of diabetes in Syria has nearly tripled, to reach a total of 11.9% in 2016 [10]. During periods of conflict, people with chronic diseases such as diabetes face numerous challenges, including inadequate access to medicines and testing supplies, and food insecurity [11].

Syria is divided into 14 administrative regions (governorates) and has a population of 17,500,657 [12, 13]. The year 2021 marks the tenth anniversary of the conflict in Syria. The conflict has resulted in one of the largest humanitarian crises in the world, with 6.6 million refugees worldwide, of whom 5.6 million are hosted in nearby countries, 6.7 million internally displaced persons, and 13.4 million people in need of humanitarian and protective assistance within the country [14, 15]. Approximately 70% of health workers have fled Syria since the start of the conflict. Furthermore, nearly 600 attacks on healthcare facilities have led to approximately 50% of them being damaged or destroyed [16]. Healthcare during humanitarian crises have typically focused on infectious diseases and other acute conditions, with lower priority given to chronic diseases. However, chronic conditions such as CVDs and diabetes are now receiving more attention as many humanitarian crises are no longer of short duration [11].

Healthcare in Syria is mainly financed by public funds, although, the costs of outpatient consultations and medicines are commonly covered by out-of-pocket expenditures [17]. There is no national health insurance that covers all inhabitants in the country. However, individuals working in public organizations, some ministries, and in professional associations are provided with health insurance [18]. This includes employees who occupy a variety of positions such as public school teachers, administrative staff, and janitors. A total of 841,852 individuals were covered by health insurance in Syria in 2019, with approximately 80% of the insured employed by the government [19]. The diagnoses and treatment of CVDs and diabetes are covered by government health insurance, however, the treatment costs of some chronic conditions such as Alzheimer’s or Parkinson’s are not. Moreover, health insurance does not cover the costs for the treatment of psychiatric illnesses or sexually transmitted diseases [20].

The prevention and control of CVDs and diabetes usually require lifestyle changes. However, pharmacological therapies are also key elements for their management [21, 22]. Diabetes, in particular, is a costly disease to manage in LMICs [23]. Globally, one in two people with type-2 diabetes have no access to the insulin they need; however, in some LMICs, this proportion is only one in six to seven patients have access to insulin [24]. The survival of 400,000 diabetic patients in Syria depends on access to insulin. Thus, due to limited supplies, approximately 60% of insulin-dependent patients are at risk [10, 11, 25]. Even when medicines are available during humanitarian crises and in conflict regions, their distribution can be challenging due to political and geographical barriers [11].

Previous studies have reported different utilization patterns for CVDs and diabetes medicines in different countries, including Iran, India, and many European countries [26, 27]. Furthermore, different patterns and higher rates of CVDs medicines prescription have been identified among males compared to females [28]. Despite the high prevalence of CVDs and diabetes in Syria, little is known about medicines utilization or prescriptions for these diseases [29]. Therefore, the aims of this study are to: 1) present the patterns and rates of dispensing medicines used for CVDs and diabetes among patients with government health insurance in Syria, and 2) examine age, sex, and regional variation in the dispensing of these medicines.

## Methods

### Data sources and patients

This study is based on outpatient medicines dispensing data from 13 out of 14 Syrian governorates. These data were only from the parts of the country under the Syrian government’s control. The government health insurance system was not functioning in the parts of the country that were out of the Syrian government’s control. Therefore, no data were available from the Ar-Raqqa governorate or some parts of other governorates, which were not under the control of the Syrian government. We used health insurance data from 81,314 adult beneficiaries employed by the Syrian government and covered by the health insurance scheme, members of professional organizations, and university students who were privately insured. The data covered a 12-month period starting from June 2018. Outpatient dispensing data included the following information regarding each dispensed medicine: product name, dose, pharmaceutical form, and administration route. Additional information shared by the health insurance company were used to identify the international non-proprietary name of each product. In addition, the data included information on the age and sex of each beneficiary, the dispensing date, prescription number, an identical number for each patient, and the name of the governorate where the medicine was dispensed.

### Data analysis

We used the World Health Organization’s (WHO’s) anatomical therapeutic chemical classification/defined daily dose (ATC/DDD) (version 2020) methodology to analyse the dispensed medicines. The defined daily dose (DDD) is “the assumed average maintenance dose per day for a drug used for its main indication in adults” [30]. The rate of dispensed medicine was expressed as the DDD per 1000 inhabitants (this corresponds to 1000 beneficiaries in our study) per day (DID). The DID is a measurement that is used in drug utilization research to control for differences or changes in population size between or within countries [31]. Medicines have been grouped using the ATC classification. In this study, CVD medicines included those from ATC groups C (cardiovascular system) and B01 (antithrombotic agents). Diabetes medicines included medicines from ATC group A10 (drugs used for diabetes). Medicine dispensing is presented according to the anatomical main group (ATC1), the therapeutic subgroup (ATC2), the pharmacological subgroup (ATC3), and the chemical substance subgroup (ATC5). We used the drug utilization 90% (DU90%) methodology to reflect the number of medicines that accounted for 90% of the dispensing rates for CVDs and diabetes medicines [32]. The number of DIDs was adjusted according to patient sex and age using the number of beneficiaries in each sex and age group which was available through the dispensing data we used in this study.

To adjust the number of DIDs according to regions or states, studies from Germany and Hungary have calculated the number of DDDs per 1000 inhabitants of each region per day [33, 34]. This analysis assumed that in these stable and high-income countries, the inhabitants of any region or state would have access to the medicines in that same region or state where they live. In Syria, the disruption of healthcare in some parts of the country due to the conflict has forced patients to flee their homes to other parts of the country to access better healthcare [35]. Our data showed that some beneficiaries had medicines for CVDs and diabetes dispensed in more than one governorate. Therefore, adjusting CVDs and diabetes medicines dispensing rates according to governorates by calculating the number of DDDs per 1000 beneficiaries (of each governorate) per day can be misleading. The CVDs and diabetes medicines dispensing rates were adjusted according to governorates by calculating the number of DDDs per 1000 medicine dispensing events per day (DDED) [36]. The medicine dispensing events here refers to the dispensing of CVDs or diabetes medicines.

### Statistical analysis

The dispensing data in this study displayed evidence of a skewed distribution of the outcome variable “rates of dispensed medicines”. Therefore, statistical significance differences between the variables of interest were assessed using nonparametric testing. The Mann-Whitney U test was used to analyze differences between the medians of the medicine dispensing rates between females and males. The Kruskal-Wallis nonparametric ANOVA was used to examine differences between the different age groups and governorates in regard to medicine dispensing rates. We considered a *p*-value of 0.05 as a cut-off value for significance in all the tests. Analyses were conducted using IBM SPSS Statistics version 25 (IBM Corp, Armonk, NY, USA).

## Results

This study used the outpatient medicine dispensing data of 81,314 adult beneficiaries who were at least 18 years of age and covered by government health insurance in Syria. The median age of the beneficiaries was 47 years (interquartile range: 37–56), and 50,673 beneficiaries (62.28%) were female and 30,671 (37.72%) were male. Table 1 lists the characteristics of the study population. The median age of the beneficiaries in Damascus, Aleppo, and Quneitra were the lowest, while the highest median age occurred in Tartous, Latakia, and Al-Suwayda (Additional file 1).

**Table 1:** Characteristics of the study population by age and sex

Age group	Female		Male		Total	
	n	%	n	%	n	%
18–29	5257	6.46	2861	3.52	8118	9.98
30–39	13,180	16.21	4603	5.66	17,783	21.87
40–49	12,372	15.22	8299	10.21	20,671	25.43
50–59	13,189	16.22	8741	10.75	21,930	26.97
60–69	5733	7.05	4311	5.30	10,044	12.35
70≤	912	1.12	1856	2.28	2768	3.40
**Total**	50,643	62.28	30,671	37.72	81,314	100

Of the total number of beneficiaries, 46,281 (56.92%) received at least one medicine in the 12-month period from June 2018 to May 2019. The total number of DIDs dispensed for these patients was 591.21 DIDs. Considering the number of DIDs, medicines in anatomical main group C (the cardiovascular system) of the ATC classification system were the most dispensed (35.78%), followed by those in group A (the alimentary tract and metabolism) (22.43%) (Additional file 2).

A total of 14,523 patients (17.84%) received 302.09 DIDs of medicines used for cardiovascular system (C) and antithrombotic agents (B01). Medicines in the ATC therapeutic subgroup B01 (antithrombotic agents) were the most dispensed medicines (90.54 DIDs) followed by C10 (lipid-modifying agents; 83.64 DIDs) and C09 (renin-angiotensin system agents; 64.48 DIDs) (Table 2).

**Table 2:** Outpatient dispensing rates of CVDs medicines according to the therapeutic subgroup (ATC2)

	Therapeutic subgroup (ATC2)	DID^**a**^		patients	
		n	%	n	%
**Cardiovascular diseases medicine**	Antithrombotic agents **(B01)**	90.54	15.31	9433	11.60
	Lipid-modifying agents **(C10)**	83.64	14.15	8765	10.78
	Renin-angiotensin system agents **(C09)**	64.48	10.91	8377	10.30
	Beta-blocking agents **(C07)**	30.48	5.16	6543	8.05
	Calcium-channel blockers **(C08)**	14.35	2.43	2031	2.48
	Cardiac therapy **(C01)**	9.26	1.57	1438	1.77
	Diuretics **(C03)**	8.12	1.37	1336	1.64
	Antihypertensives **(C02)**	1.00	0.17	334	0.41
	Peripheral vasodilators **(C04)**	0.18	0.03	230	0.28
	Vasoprotectives **(C05**)	0.04	0.01	2441	3.00
	**Total**	302.09	51.11	14,532^b^	17.84^b^

^a^Outpatient medicines dispensing according to the therapeutic subgroup (ATC2) is expressed as the number of defined daily doses (DDDs) per 1000 people per day (DID)

^b^Some patients dispensed different CVDs medicines. To avoid counting these patients more than once, we considered the identical number for each patient while calculating the total number and percentage of patients

A total of 4466 patients (5.49%) received 35.66 DIDs of diabetes medicines (medicines used in diabetes: A10). Medicines in the pharmacological subgroup A10B (blood glucose lowering drugs, excluding insulin; 34.70 DIDs) were the most dispensed among diabetes medicines, while 0.96 DID of the A10A group (insulin and analogues) were dispensed for diabetes patients (Table 3).

**Table 3:** Outpatient dispensing rates of diabetes medicines according to the pharmacological subgroup (ATC3) including insulin (ATC5)

**Therapeutic subgroup (ATC3)**	**DID**^**a**^		**Patients**	
	**n**	**%**	**n**	**%**
Insulin and analogues **(A10A)**	0.96	0.16	117	0.14
Blood glucose lowering drugs excluding insulins **(A10B)**	34.70	5.87	4413	5.43
**Total**	35.66	6.03	4466^b^	5.49^b^
**Medicine (ATC5)**	**DID**^**a**^		**Patients**	
	**n**	**%**	**n**	**%**
Insulin (human) intermediate- or long-acting combined with fast-acting (A10AD01)	0.55	0.09	67	0.08
Insulin aspart (A10AD05)	0.18	0.03	25	0.03
Insulin (human) fast acting (A10AB01)	0.09	0.02	10	0.01
Insulin lispro (A10AD04)	0.08	0.01	12	0.01
Insulin glargine (A10AE04)	0.04	0.01	9	0.01
Insulin (human) intermediate acting (A10AC01)	0.02	0.004	4	0.005
**Total**	0.96	0.16	117^b^	0.14^b^

^a^Dispensing rates of diabetes medicines including insulin are expressed as the number of defined daily doses per 1000 people per day (DID)

^b^Some patients dispensed different diabetes medicines. To avoid counting these patients more than once, we considered the identical number for each patient while calculating the total number and percentage of patients

Acetylsalicylic acid was the most dispensed CVDs medicine (60.84 DIDs) followed by rosuvastatin (47.56 DIDs) and clopidogrel (23.03 DIDs). Thirty medicines accounted for 272.92 DIDs, which were 90% of the total DIDs of the dispensed CVDs medicines. Metformin was the most dispensed diabetes medicine (8.29 DIDs) followed by gliclazide (7.44 DIDs) and the fixed dose combination of metformin and sulfonylureas (6.82 DIDs). Eight medicines accounted for 32.76 DIDs, which were 91.86% of the total DIDs of the dispensed diabetes medicines (Additional file 3).

Of the 4466 patients who received diabetes medicines, 117 (2.62%) received 0.96 DID of insulin and analogues. Approximately 30% of the dispensed insulins were insulin analogues. Human insulin (intermediate- or long-acting combined with fast-acting insulin) was the most dispensed (0.55 DIDs) followed by insulin aspart (0.18 DIDs) and fast-acting human insulin (0.9 DIDs) (Table 3).

**Fig. 1:**
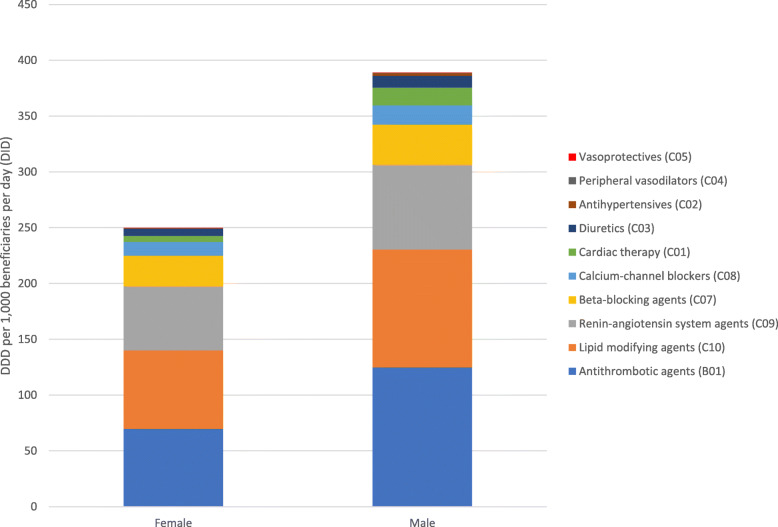
Adjusted CVDs medicines dispensing rates (DID) according to the therapeutic subgroup (ATC2) by sex

We found that 16.12% (8384) of female beneficiaries and 19.66% (6141) of male beneficiaries received outpatient CVDs medicines. The adjusted DID rates of CVDs medicines were higher among male patients (388.80 DIDs) than female patients (249.59 DIDs) and this difference was statistically significant (*p* < 0.001). Among both females and males, the CVDs medicines in the ATC therapeutic subgroup B01 (antithrombotic agents) were the most dispensed followed by C10 (lipid-modifying agents) and C09 (renin-angiotensin system agents) (Fig. 1). We found that 4.91% (2489) of the female beneficiaries and 6.45% (1977) of the male beneficiaries received outpatient diabetes medicines. The adjusted DID rates of diabetes medicines were higher among males (45.98 DIDs) than females (29.42 DIDs) and this difference was statistically significant (*p* < 0.001). The adjusted DID rates for insulins were also higher among male patients in comparison to females (1.14 DIDs and 0.85 DIDs, respectively).

**Fig. 2:**
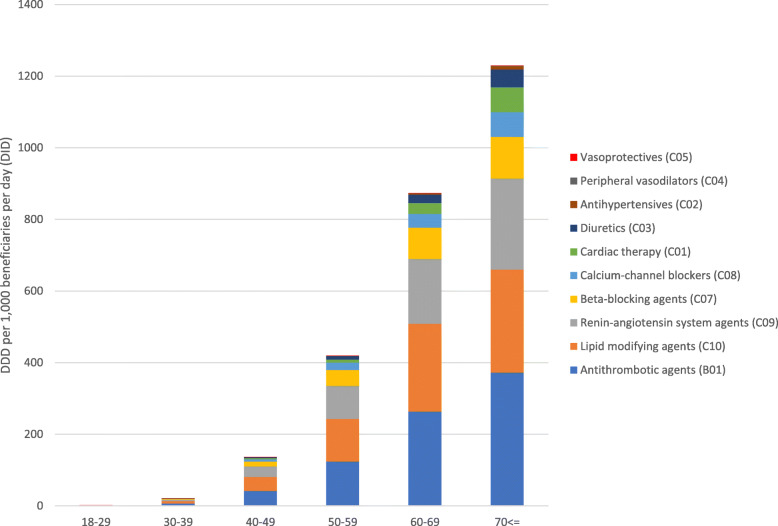
Adjusted cardiovascular system medicines dispensing rates (DDD) among adults with health insurance in Syria by age groups

For beneficiaries in the age group 18–29, 3.76% received CVDs medicines. The proportion of patients who received CVDs medicines increased with age and was 50.53% in the 70 ≤ age group. The adjusted DID rates of CVDs medicines were the lowest in the 18–29 age group (1.80 DIDs) and increased to 1229.04 DIDs in the 70 ≤ age group. This difference was statistically significant (*p* < 0.001). The CVDs medicines in the ATC therapeutic subgroup B01 (antithrombotic agents) were the most dispensed CVDs medicines followed by C10 (lipid-modifying agents) and C09 (renin-angiotensin system agents) in all age groups except in the 30–39 age group where the ATC therapeutic subgroup C10 (lipid-modified agents) were the most dispensed, followed by B01 (antithrombotic agents) (Fig. 2).

**Fig. 3:**
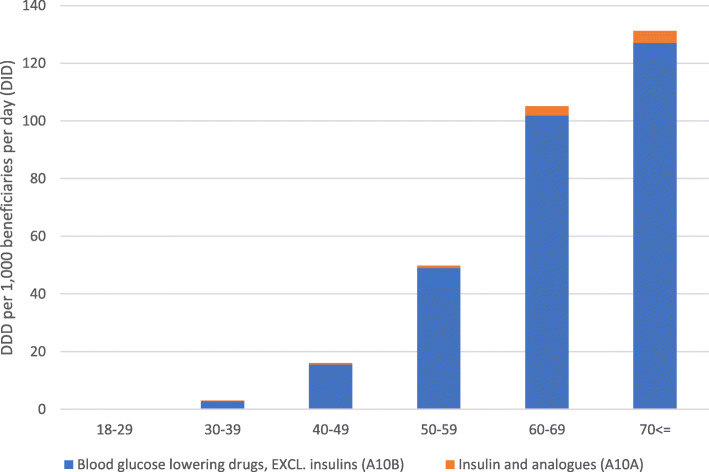
Adjusted diabetes medicines dispensing rates (DID) according to the pharmacological subgroup by age groups

Of the beneficiaries in the age group 18–29, 0.58% received diabetes medicines. The proportion of patients who received diabetes medicines increased with age to 21.46% in the 70 ≤ age group. The adjusted DID rates of diabetes medicines were the lowest among patients in the 18–29 age group (0.28 DIDs) and increased to 131.34 DIDs in the 70 ≤ age group (Fig. 3). This difference was statistically significant (*p* < 0.001).

**Fig. 4:**
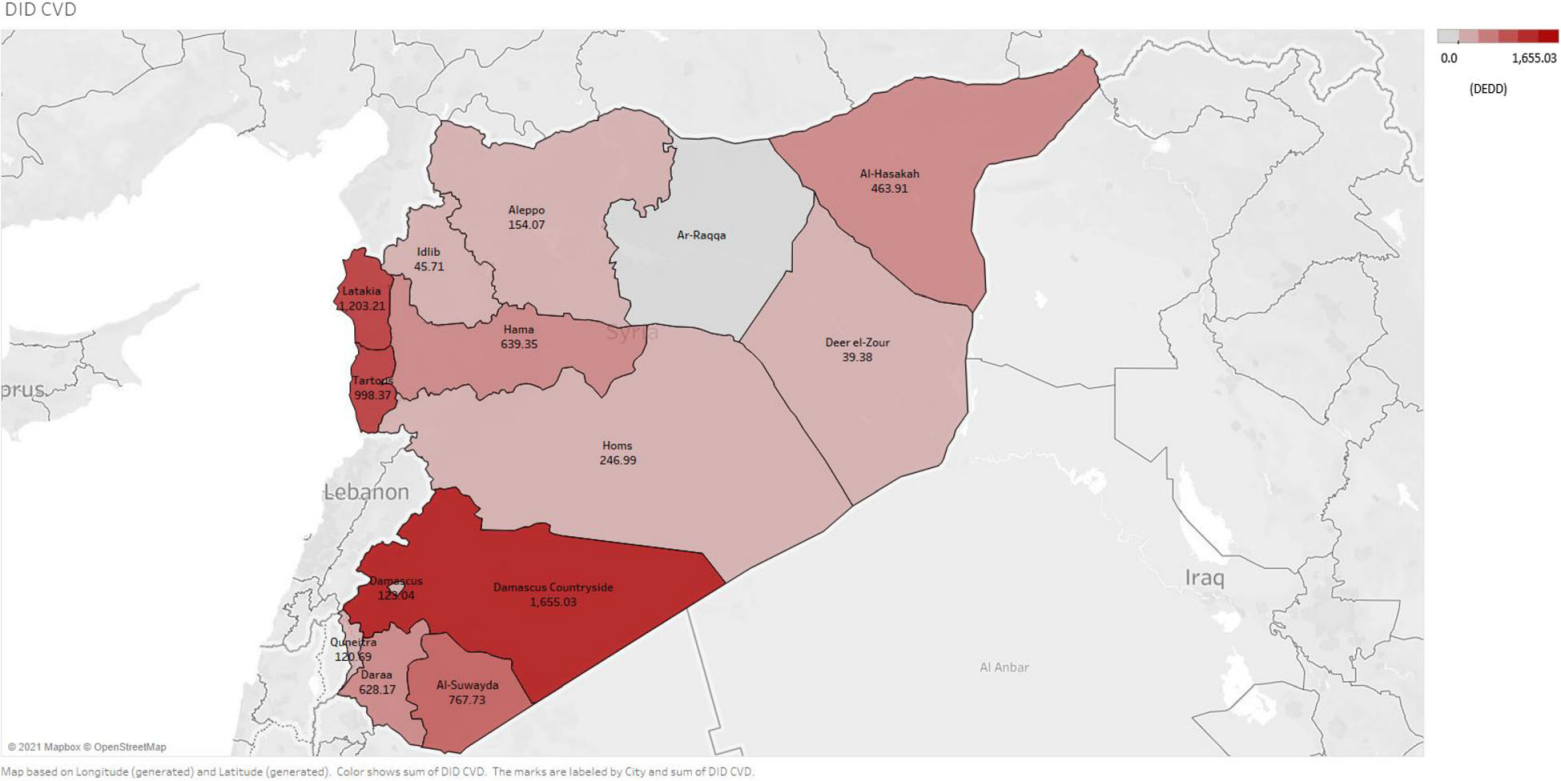
Adjusted CVDs medicines dispensing rates (DDED) among adults with health insurance in Syria by governorate [37]

We found a statistically significant difference (*p* < 0.001) in CVDs medicine dispensing rates between Syria’s different governorates. According to the number of DDEDs in each governorate, the Damascus countryside (1655.03 DDED), Latakia (1203.21 DDEDs), and Tartous (998.37 DDEDs) had the highest CVDs medicine dispensing rates, while Deer el-Zour (39.38 DDED), Idlib (45.71 DDEDs), and Quneitra (120.69 DDEDs) had the lowest CVDs medicine dispensing rates (Fig. 4).

**Fig. 5:**
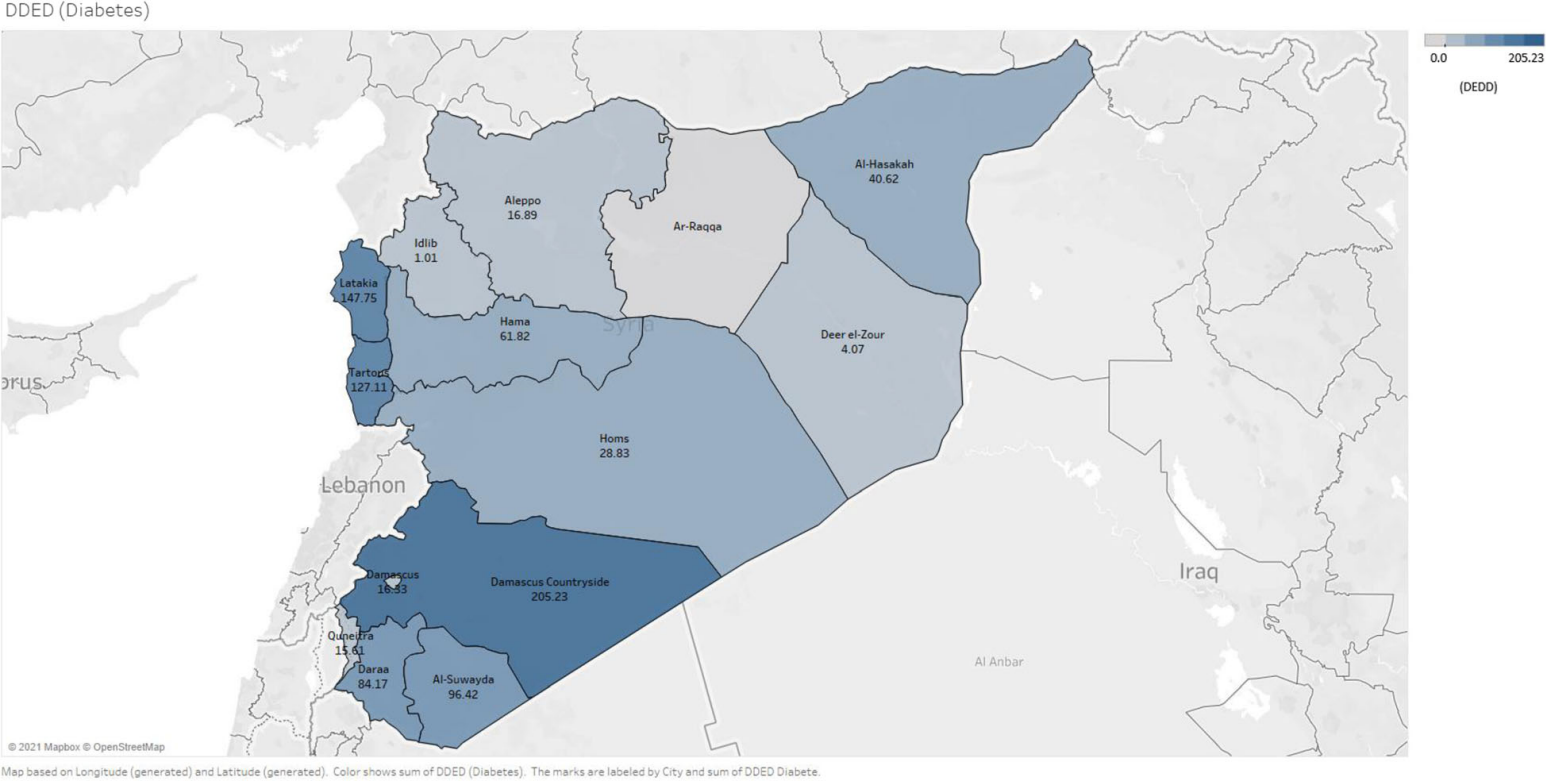
Adjusted diabetes medicines dispensing rates (DDED) among adults with health insurance in Syria by governorate [37]

In terms of diabetes medicine dispensing rates, the difference between Syria’s different governorates was statistically significant (*p* < 0.001). The Damascus countryside (205.23 DDEDs), Latakia (147.75 DDEDs), and Tartous (127.11 DDEDs) had the highest diabetes medicine dispensing rates, while Idlib (1.01 DEDDs), Deer el-Zour (4.07 DDEDs) and Quneitra (15.61 DDEDs) had the lowest diabetes medicine dispensing rates (Fig. 5). The highest insulin dispensing rates were recorded in the Damascus countryside (14.32 DDEDs), Latakia (6.85 DDEDs), and Al-Suwayda (6.74 DDEDs). The insurance data did not record insulin dispensing in the governorates of Al-Hasakah, Idlib, Deer el-Zour, or Daraa (Additional file 4).

## Discussion

To the best of our knowledge, this is the first study to report CVDs and diabetes medicine dispensing at the population level in Syria using health insurance data from a large sample (81,314 beneficiaries) over a 12-month period. This study contributes to our knowledge regarding treatments for two common NCDs in Syria: CVDs and diabetes. Our analysis yielded three key findings: 1) CVDs and diabetes medicine dispensing rates were low during the study period and included very low rates of insulin dispensing; 2) there were lower dispensing rates of CVDs and diabetes medicines among female beneficiaries compared to male beneficiaries; and 3) there were higher rates of CVDs and diabetes medicines dispensed in governorates that were completely or mostly controlled by the government and very low to no dispensing of insulin in some governorates that were partly controlled by the Syrian government.

The rates for dispensing cardiovascular system medicines (C) were the highest in comparison to the other medicine groups. This can be explained by the high rate of CVDs in Syria [38]. CVDs medicine dispensing rates in our study were, however, low compared to many other countries. A study from Australia reported higher rates of dispensed CVDs medicines (566.00 DIDs) [39], and a study from Serbia reported higher dispensing rates for some antihypertensives (283 DIDs) compared to the dispensing rates of antihypertensives in our study [40]. These differences in medicine use between countries may be the result of differences in the age and sex distribution of the populations, differences in the prevalence of high blood pressure and cholesterol, and variation in clinical practices [41]. Other reasons for the low dispensing rates of CVDs medicines found in this study could be associated with the ongoing conflict in Syria. Access to medicines, including CVDs medicines, can be affected by several barriers, including travel bans and checkpoints during conflict [42].

The dispensing rate of diabetes medicines in Syria was quite low compared to those in other countries. A study that reported on the consumption of antidiabetic medicine in the 28 countries of the Organization for Economic Co-operation and Development (OECD) found that the average rate of diabetes medicine consumption was 68 DIDs, which is nearly double the rate found in our study. Similarly, in neighbouring Turkey the consumption rate of diabetes medicine is 73 DIDs, which is more than double the rate we found in our study [43]. Another study reported that the rate of diabetic medicine consumption in Iran in 2012 was also low in comparison to other countries (33.54 DIDs) [26]. However, this rate may have increased in the years 2018–2019 to exceed the rate of diabetes medicine consumption in our study, as the authors of that report indicated an ongoing increase in diabetes medicine rates with time in Iran. The low dispensing rates of diabetic medicine that we found could be related to a potentially high number of undiagnosed diabetes in Syria. The WHO has reported that four in five undiagnosed diabetic patients live in LMICs [44]. One study has reported that diabetes was well controlled in only 16.7% of type-2 diabetes patients in Aleppo in 2011—before the start of the conflict [45]. Since there is an evidence on the low number of controlled diabetes cases it would be expected that there is a low rate of diabetes medicines utilization. In addition, the low dispensing rates of diabetes medicines may be explained by factors related to the conflict situation in Syria. A study conducted among a group of diabetes patients in main public hospitals in Damascus during the Syrian crisis reported that 41.2% of the patients had stopped their medicines for at least 1 month over the course of 7 years of conflict, and 74.8% of these patients attributed stopping their therapy to the unavailability of medicines. Furthermore, approximately half of the patients had to change their medicines brand names because many pharmaceutical companies closed due to the conflict. In addition, half of the diabetic patients struggled to reach a healthcare center [46]. Moreover, food insecurity in countries affected by conflict limits the possibility of diabetes patients to adhere to recommended diets, which would present an obstacle to maintaining treatment as diabetes medicines must be taken with food [47].

Compared to the insulin dispensing rate in our study, higher rates of insulin prescriptions in other counties has been reported. For instance, a study from Albania reported a 5.64 DIDs insulin outpatient prescription rate [48], while Iran reported higher insulin consumption rates in 2012 (5.73 DIDs) [26]. A study from Portugal reported an insulin dispensing rate of 15.1 DIDs in 2014. In addition to the factors we have previously mentioned to explain the low dispensing rates of diabetes medicine, specific factors related to insulin could also explain the low rates. The limited availability of insulin due to the conflict in the country may be another factor. Approximately 60% of insulin-dependent Syrians are at risk due to limited supplies [11, 25]. Furthermore, insulin requires cold-chain transportation and the maintenance of temperatures between 2 °C and 8 °C. The storage of insulin by patients is an additional challenge as the lack of refrigeration is common due to frequent energy cuts [11]. In addition, our data may not include all of the insulin given to insulin-dependent diabetic patients, as they could have received insulin through other channels such as humanitarian organizations—the WHO is now the main supplier of insulin in Syria [25]. Poor adherence to insulin caused by injection phobia among some patients has been recorded in other studies [47]. This can also further explain the very low rates of insulin dispensing in our study.

CVDs medicine dispensing rates were significantly higher among males than females. Other studies have reported higher rates of hypertension among middle-aged females (35 to 65 years) than males in Syria and other countries of the Eastern Mediterranean region and North Africa [49]. Moreover, women of Arab ethnicity present with coronary artery disease 10 years earlier than those from Europe or East Asia [50]. Obesity, a significant risk factor of CVDs, also has a higher prevalence among females in Eastern Mediterranean region countries, including Syria, compared to males in these countries [38, 51, 52]. Cultural and religious norms, as well as circumstances related to the conflict and lack of security, restrict women from sports and limit physical activity. These factors may also contribute to the CVDs burden among women in Syria and the region [50]. The low dispensing rates of CVDs medicines among females in our study contrasts with other studies that have reported high rates of CVDs in females in Syria. This suggests that CVDs were undertreated among females in our study population. Despite being the leading cause of death among women globally, CVDs in women are still understudied, underdiagnosed, and undertreated [53]. There is a common misperception that CVDs affect men more than women, and this may contribute to the suboptimal treatment of CVDs among women [28]. Similar to CVDs, the dispensing rates of diabetes medicines, including insulin dispensing rates, were significantly higher among males than females; however, the WHO has reported a higher prevalence of diabetes among females in Syria than males [25]. This result indicates the undertreatment of diabetes among females in our study. Other studies have reported poorer control of type-2 diabetes among women than men [54–56]. Furthermore, the conflict situation in Syria may have contributed to women’s vulnerability in comparison to men. This crisis has restricted women’s movement more than men and limited their access to healthcare, including diabetic care [11, 57]. Unsurprisingly, the dispensing rates of CVDs and diabetes medicines increased with increasing age, which is due to the increasing prevalence of CVDs and diabetes with increasing age [58, 59].

There was a significant difference in the dispensing rates of CVDs and diabetes medicines between the different governorates in Syria. The Damascus countryside, Latakia, and Tartous had the highest dispensing rates for CVDs and diabetes medicines, while Idlib, Deer el-Zour, and Quneitra had the lowest rates. The major areas of the three governorates with the highest CVDs and diabetes medicines dispensing rates (Damascus countryside, Latakia, and Tartous) were under the Syrian government’s control. However, while the major areas of Deer el-Zour and Idlib were out of the Syrian government’s control, they were affected by the armed conflict during the study period [60]. This regional variation may be related to the conflict situation that has rendered services damaged or unavailable. There is an uneven distribution of healthcare services, including medicines, across geographical regions [61, 62]. Through internal displacement, the conflict has also contributed to regional variation in Syrian healthcare services. The disruption of healthcare in some parts of Syria has forced patients with chronic diseases, especially older individuals, to flee their homes to other parts of the country to access better healthcare [35]. Our data did not record insulin dispensing in Al-Hasakah, Idlib, Deer el-Zour, and Daraa. Being a cold chain product, delivery of insulin in these regions may have been extremely challenging. The distribution of essential medicines, including insulin even when it was available, can be complex due to geographical and political barriers as many parts of the country, especially those remote from the regime hubs, are controlled by opposing forces to the Syrian government [11].

### Strengths and limitations

The study’s limitations principally arose from the data that was used, as it did not provide the diagnoses underlying the prescribed medicines. The generalizability is also limited as our data only included people with government health insurance. The majority of our study population were employed by the Syrian government. Government employees in Syria belong usually to the country’s middle-income class [63]. The study did not include people living in areas of Syria that were out of government control. In particular, the number of insured people in Deer el-Zour and Idlib was significantly lower than other governorates because large areas of these regions were beyond the Syrian government’s control. Further studies are necessary to reach those regions. Our data did not necessarily include all the diabetes medicines used by diabetic patients because some might receive these medicines, especially insulin, through other sources such as humanitarian organizations. Despite these limitations, our study represents an essential step towards understanding medicine use for NCDs such as CVDs and diabetes in a country plagued by an ongoing conflict since 2011. Our research also provides a picture of CVD and diabetes management in a large sample that is diverse in terms of age and sex and included data on medicine use in 13 out of 14 Syrian governorates. Finally, reporting medicine dispensing rates and patterns using ATC/DDD methodology enables the comparison of medicine dispensing at the international level [64].

## Conclusions

This study presents the first estimates of CVDs and diabetes medicines dispensing rates and patterns at population level in Syria using health insurance data from a large sample (81,314 beneficiaries) over 12 months. Our study demonstrated significant difference in CVDs and diabetes medicines dispensing rates between male and female beneficiaries. This study also showed significant regional variation in CVDs and diabetes medicines dispensing with low CVDs and diabetes medicines dispensing rates and very low to no dispensing of insulin in some governorates that the Syrian government partly controlled. Additional efforts are needed to raise awareness about the prevention and management of CVDs and diabetes, especially among females in Syria including cultural issues that might influence access to healthcare services. In addition, the growing importance of NCDs during humanitarian crises should be recognized by all healthcare providers. Humanitarian healthcare workers should be trained to handle CVDs and diabetes emergencies. There is a crucial need to address the political and geographical challenges caused by the conflict in Syria, limiting access to CVDs and diabetes medicines in some regions in Syria. It is the responsibility of the Syrian government and the other different actors across the country to ensure continuous access to healthcare services, including essential medicines, to the population in the different regions of Syria. We encourage further research that would address CVDs, and diabetes medicines use in the areas beyond the control of the Syrian government and among the groups of the population that are not covered by the government health insurance. Future research should give more attention to improving the prevention and management of NCDs during humanitarian crises.

## Supplementary Information

**Additional file 1.** Median age of beneficiaries by governorate.

**Additional file 2.** Outpatient medicines dispensing rates according to the anatomical main group (ATC1).

**Additional file 3.** DU90% of CVDs and diabetes medicines.

**Additional file 4.** Adjusted insulin dispensing rates (DDED) among adults with health insurance in Syria by governorate.

## Data Availability

The datasets generated and analysed during the current study are not publicly available due a request from the data provider but are available from the corresponding author on reasonable request.

## Abbreviations

ATC: Anatomical Therapeutic Chemical Classification
CVD: Cardiovascular Disease
DDD: Defined Daily Dose
DDED: Number of Defined Daily Dose per 1000 Medicine Dispensing Events per Day
DID: Defined Daily Dose per 1000 beneficiaries per day
DU90%: Drug Utilization 90%
LMICs: Low- and Middle-Income Countries
NCD: Non-Communicable Disease
OECD: Organization for Economic Co-operation and Development
WHO: World Health Organization

## References

[1] World Health Organization (2021). Noncommunicable diseases.

[2] Devi S (2021). Aid agencies turn attention to diabetes. Lancet.

[3] Kankeu HT, Saksena P, Xu K, Evans DB (2013). The financial burden from non-communicable diseases in low- and middle-income countries: a literature review. Health Res Policy Syst.

[4] World Health Organization: Global action plan for the prevention and control of noncommunicable diseases 2013-2020. Available from: https://apps.who.int/iris/bitstream/handle/10665/94384/9789241506236_eng.pdf?sequence=1&isAllowed=y. Accessed 2 June 2021.

[5] Chan JCN, Lim L-L, Wareham NJ, Shaw JE, Orchard TJ, Zhang P (2020). The lancet commission on diabetes: using data to transform diabetes care and patient lives. Lancet.

[6] World Health Organization: Cardiovascular diseases (CVDs). Available from: https://www.who.int/news-room/fact-sheets/detail/cardiovascular-diseases-(cvds). Accessed 12 June 2021.

[7] Ansbro É, Homan T, Prieto Merino D, Jobanputra K, Qasem J (2021). Clinical outcomes in a primary-level non-communicable disease programme for Syrian refugees and the host population in Jordan: a cohort analysis using routine data. PLoS Med.

[8] World Health Organization (2011). Noncommunicable diseases country profiles.

[9] World Health Organization: Noncommunicable diseases country profiles. Available from: file:///C:/Users/asus/AppData/Local/Temp/9789241514620-eng.pdf. Accessed 7 June 2021.

[10] World Health Organization: Diabetes country profiles. Available from: https://www.who.int/diabetes/country-profiles/syr_en.pdf. Accessed 3 June 2021.

[11] Khan Y, Albache N, Almasri I, Gabbay RA (2019). The Management of Diabetes in conflict settings: focus on the Syrian crisis. Diabetes Spectrum.

[12] The World Bank: Population, total - Syrian Arab Republic. Available from: https://data.worldbank.org/indicator/SP.POP.TOTL?locations=SY. Accessed 3 Sept 2021.

[13] European Committee of the Regions: Syria Policy Area. Available from: https://portal.cor.europa.eu/divisionpowers/Pages/Syria-Introduction.aspx. Accesses 3 Sept 2021.

[14] UNICEF (2020). Syria Crisis. Humanitarian Situation Report.

[15] UNHCR (2021). Syria emergency.

[16] Devi S (2021). Health in Syria: a decade of conflict. Lancet.

[17] Sen K, Al Faisal W (2012). Syria neoliberal reforms in health sector financing: embedding unequal access?. Soc Med.

[18] Schwefel D. Towards a national health insurance system in Syria. http://detlef-schwefel.de/246-Schwefel-Syria-health-insurance.pdf. Accessed 7 June 2021.

[19] Syrian Insurance Supervisory Commission (2019). Syrian insurance sector report for.

[20] Syrian General Association for Insurance: Health Insurance Guide [https://www.sic-eclaim.com/Assets/SIC/Docs/HealthcareInsuranceGuide.pdf].

[21] Wirtz VJ, Kaplan WA, Kwan GF, Laing RO (2016). Access to medications for cardiovascular diseases in low- and middle-income countries. Circulation.

[22] Nathan DM, Buse JB, Davidson MB, Ferrannini E, Holman RR, Sherwin R, Zinman B (2009). Medical management of hyperglycemia in type 2 diabetes: a consensus algorithm for the initiation and adjustment of therapy: a consensus statement of the American Diabetes Association and the European Association for the Study of diabetes. Diabetes Care.

[23] Moucheraud C, Lenz C, Latkovic M, Wirtz VJ (2019). The costs of diabetes treatment in low- and middle-income countries: a systematic review. BMJ Glob Health.

[24] Basu S, Yudkin JS, Kehlenbrink S, Davies JI, Wild SH, Lipska KJ (2019). Estimation of global insulin use for type 2 diabetes, 2018–30: a microsimulation analysis. Lancet Diabet Endocrinol.

[25] World Health Organization: WHO helps diabetes patients in Syria. Available from: https://www.who.int/news-room/feature-stories/detail/who-helps-diabetes-patients-in-syria. Accessed 7 June 2021.

[26] Sarayani A, Rashidian A, Gholami K (2014). Low utilisation of diabetes medicines in Iran, despite their affordability (2000-2012): a time-series and benchmarking study. BMJ Open.

[27] Jarari N, Rao N, Peela JR, Ellafi KA, Shakila S, Said AR (2015). A review on prescribing patterns of antihypertensive drugs. Clin Hypertens.

[28] Zhao M, Woodward M, Vaartjes I, Millett ERC, Klipstein-Grobusch K, Hyun K (2020). Sex differences in cardiovascular medication prescription in primary care: a systematic review and Meta-analysis. J Am Heart Assoc.

[29] Akl C, Akik C, Ghattas H, Obermeyer CM (2020). The cascade of care in managing hypertension in the Arab world: a systematic assessment of the evidence on awareness, treatment and control. BMC Public Health.

[30] WHO Collaborating Centre for Drug Statistics Methodology: Anatomical Therapeutic Chemical (ATC)Classification System. Available from: https://www.whocc.no/atc_ddd_index/. Accessed 1 June 2021.

[31] Elseviers M, Stichele RV, Vlahovic-Palcevski V (2016). Drug utilization research: methods and applications.

[32] Bergman U, Popa C, Tomson Y, Wettermark B, Einarson TR, Aberg H (1998). Drug utilization 90%--a simple method for assessing the quality of drug prescribing. Eur J Clin Pharmacol.

[33] Kern WV, With K, Nink K, Steib-Bauert M, Schröder H (2006). Regional variation in outpatient antibiotic prescribing in Germany. Infection.

[34] Matuz M, Benko R, Doro P, Hajdu E, Nagy G, Nagy E (2006). Regional variations in community consumption of antibiotics in Hungary, 1996-2003. Br J Clin Pharmacol.

[35] United Nations GA: Protection of and assistance to internally displaced persons: situation of internally displaced persons in the Syrian Arab Republic. Available from: https://www.ohchr.org/Documents/Issues/IDPersons/A_67_931Syria_report.pdf. Accessed 20 June 2021.

[36] Aljadeeah S, Wirtz VJ, Nagel E (2020). Outpatient Antibiotic Dispensing for the Population with Government Health Insurance in Syria in 2018–2019. Antibiotics.

[37] OpenStreetMap, 2021. Available from: https://www.openstreetmap.org.

[38] Al Ali R, Rastam S, Fouad FM, Mzayek F, Maziak W (2011). Modifiable cardiovascular risk factors among adults in Aleppo, Syria. Int J Public Health.

[39] Australian Institute of Health and Welfare (2017). Medicines for cardiovascular disease.

[40] Tomas A, Tomić Z, Milijasević B, Ban M, Horvat O, Vukmirović S (2016). Patterns of prescription antihypertensive drug utilization and adherence to treatment guidelines in the city of Novi Sad. Vojnosanit Pregl.

[41] Wilkins E, Wilson L, Wickramasinghe K, Bhatnagar P, Leal J, Luengo-Fernandez R, Burns R (2017). European cardiovascular disease statistics.

[42] Leyh BM, Gispen ME (2018). Access to medicines in times of conflict: overlapping compliance and accountability frameworks for Syria. Health Hum Rights.

[43] OECD Health Statistics 2019: Figure 10.8. Anti-diabetic drug consumption, 2000 and 2017 (or nearest year). Available from: https://www.oecd-ilibrary.org/sites/43146d4b-en/index.html?itemId=/content/component/43146d4b-en. Accessed 15 June 2021.

[44] World Health Organization: Changing the game to improve availability and affordability of quality-assured insulin and associated devices. Available from: https://www.who.int/news/item/25-09-2020-changing-the-game-to-improve-availability-and-affordability-of-quality-assured-insulin-and-associated-devices. Accessed 20 June 2021.

[45] Albache N, Al Ali R, Rastam S, Fouad FM, Mzayek F, Maziak W (2010). Epidemiology of type 2 diabetes mellitus in Aleppo, Syria. J Diabetes.

[46] Hamzeh A, Almhanni G, Aljaber Y, Alhasan R, Alhasan R, Alsamman MI (2019). Awareness of diabetes and diabetic retinopathy among a group of diabetic patients in main public hospitals in Damascus, Syria during the Syrian crisis. BMC Health Serv Res.

[47] Murphy A, Biringanine M, Roberts B, Stringer B, Perel P, Jobanputra K (2017). Diabetes care in a complex humanitarian emergency setting: a qualitative evaluation. BMC Health Serv Res.

[48] Kakariqi L (2016). Trends in prescribing and utilization of Antidiabetic drugs in primary health Care in Albania during 2004-2014. IJSM.

[49] Akl C, Akik C, Ghattas H, Obermeyer CM (2017). Gender disparities in midlife hypertension: a review of the evidence on the Arab region. Womens Midlife Health.

[50] Vogel B, Acevedo M, Appelman Y, Bairey Merz CN, Chieffo A, Figtree GA (2021). The lancet women and cardiovascular disease commission: reducing the global burden by 2030. Lancet.

[51] Djalalinia S, Saeedi Moghaddam S, Sheidaei A, Rezaei N, Naghibi Iravani SS, Modirian M (2020). Patterns of obesity and overweight in the Iranian population: findings of STEPs 2016. Front Endocrinol.

[52] Weiderpass E, Botteri E, Longenecker JC, Alkandari A, Al-Wotayan R, Al Duwairi Q (2019). The prevalence of overweight and obesity in an adult Kuwaiti population in 2014. Front Endocrinol.

[53] Mocumbi AO (2021). Women's cardiovascular health: shifting towards equity and justice. Lancet.

[54] Tang YH, Pang SMC, Chan MF, Yeung GSP, Yeung VTF (2008). Health literacy, complication awareness, and diabetic control in patients with type 2 diabetes mellitus. J Adv Nurs.

[55] Chiu C-J, Wray LA (2011). Gender differences in functional limitations in adults living with type 2 diabetes: biobehavioral and psychosocial mediators. Ann Behav Med.

[56] Shalev V, Chodick G, Heymann AD, Kokia E (2005). Gender differences in healthcare utilization and medical indicators among patients with diabetes. Public Health.

[57] Akik C, Semaan A, Shaker-Berbari L, Jamaluddine Z, Saad GE, Lopes K (2020). Responding to health needs of women, children and adolescents within Syria during conflict: intervention coverage, challenges and adaptations. Confl Health.

[58] Suastika K, Dwipayana P, Siswadi M, Tuty RA. Age is an important risk factor for type 2 Diabetes Mellitus and Cardiovascular Diseases. In: Chackrewarthy S, editor. Glucose Tolerance. Croatia: InTech; 2012.

[59] Rodgers JL, Jones J, Bolleddu SI, Vanthenapalli S, Rodgers LE, Shah K (2019). Cardiovascular risks associated with gender and aging. J Cardiovasc Dev Dis.

[60] European Asylum Support Office: Syria Security Situation. Available from: https://coi.easo.europa.eu/administration/easo/PLib/11_2019_EASO_COI_Report_Syria_Security_situation.pdf. Accessed on 08 Jun 2021.

[61] Ben Taleb Z, Bahelah R, Fouad FM, Coutts A, Wilcox M, Maziak W (2015). Syria: health in a country undergoing tragic transition. Int J Public Health.

[62] Kherallah M, Alahfez T, Sahloul Z, Eddin KD, Jamil G (2012). Health care in Syria before and during the crisis. Avicenna J Med.

[63] Syrian Economic Sciences Society: Employment and Livelihood Support in Syria: A Study Conducted for UNDP Syria by the Syrian Economic Sciences Society. Available from: file:///C:/Users/asus/AppData/Local/Temp/Employment%20and%20Livelihoods%20study_English.pdf. Accessed 11 July 2021.

[64] WHO Collaborating Center for Drug Statistics Methodology: Purpose of the ATC/DDD system. Available from https://www.whocc.no/atc_ddd_methodology/purpose_of_the_atc_ddd_system/. Accessed 20 June 2021.

