# Supplementary material for: Outpatient cardiovascular diseases and diabetes medicines dispensing in the population with government health insurance in Syria between 2018 and 2019: a retrospective analysis
Source: BMC Health Serv Res. 2021 Oct 13;21:1088. doi: 10.1186/s12913-021-07124-6 (PMC8515648; doi:10.1186/s12913-021-07124-6)
Supplement: Supplementary file 1 — Additional file 1. Median age of beneficiaries by governorate. [file 12913_2021_7124_MOESM1_ESM.docx]

**Additional file 1.** Median age of beneficiaries by governorate.

| **Governorate** | **Median age (interquartile range)** |
| --- | --- |
| Aleppo | 45 (30-53) |
| Al-Hasakah | 49 (41-57) |
| Al-Suwayda | 62 (54-69) |
| Damascus | 43 (33-52) |
| Damascus Countryside | 58 (49-64) |
| Daraa | 58 (53-64) |
| Deer el-Zour | 54 (45-62) |
| Hama | 47 (40-55) |
| Homs | 47 (37-55) |
| Idlib | 52 (44-57) |
| Latakia | 63 (58-67) |
| Quneitra | 47 (38-57) |
| Tartous | 64 (60-69) |
