# Supplementary material for: Outpatient cardiovascular diseases and diabetes medicines dispensing in the population with government health insurance in Syria between 2018 and 2019: a retrospective analysis
Source: BMC Health Serv Res. 2021 Oct 13;21:1088. doi: 10.1186/s12913-021-07124-6 (PMC8515648; doi:10.1186/s12913-021-07124-6)
Supplement: Supplementary file 2 — Additional file 2. Outpatient medicines dispensing rates according to the anatomical main group (ATC1). [file 12913_2021_7124_MOESM2_ESM.docx]

**Additional file 2****.** Outpatient medicines dispensing rates according to the anatomical main group (ATC1).

| **Anatomical main group (ATC1)** | **DID*** | | **Patients** | |
| --- | --- | --- | --- | --- |
|  | n | % | n | % |
| Cardiovascular system **(C)** | 211.56 | 35.78 | 14,192 | 17.45 |
| Alimentary tract and metabolism **(A)** | 132.63 | 22.43 | 29,363 | 36.11 |
| Blood and blood forming organs **(B)** | 108.94 | 18.43 | 14,869 | 18.29 |
| Musculo-skeletal system **(M)** | 43.71 | 7.39 | 33,153 | 40.77 |
| Respiratory system **(R)** | 38.64 | 6.54 | 22,981 | 28.26 |
| Anti-infectives for systematic use **(J)** | 20.99 | 3.55 | 33,720 | 41.47 |
| Systemic hormonal preparations, EXCL. sex hormones and insulin **(H)** | 19.28 | 3.26 | 8,369 | 10.29 |
| Genito-urinary system and sex hormones **(G)** | 6.23 | 1.05 | 5,619 | 6.91 |
| Nervous system **(N)** | 5.76 | 0.98 | 15,126 | 18.60 |
| Antiparasitic products **(P)** | 1.51 | 0.26 | 10,239 | 12.59 |
| Sensory organs | 1.05 | 0.18 | 4,003 | 4.92 |
| Antineoplastic and immunomodulating agents **(L)** | 0.76 | 0.13 | 124 | 0.15 |
| Dermatologicals **(D)** | 0.15 | 0.02 | 7,686 | 9.45 |
| Various **(V)** | 0** | 0 | 30 | 0.04 |
| **Total** | 591.21 | 100 | 46,281*** | 56.92*** |

* Medicines dispensing according to the anatomical main group (ATC1) is expressed as the number of defined daily doses (DDDs) per 1,000 beneficiaries per day (DID).

**No defined daily doses were assigned for medicines in this group.

***Some patients dispensed different medicines. To avoid counting these patients more than once, we considered the identical number for each patient while calculating the total number and percentage of patients.
