# Supplementary material for: Outpatient cardiovascular diseases and diabetes medicines dispensing in the population with government health insurance in Syria between 2018 and 2019: a retrospective analysis
Source: BMC Health Serv Res. 2021 Oct 13;21:1088. doi: 10.1186/s12913-021-07124-6 (PMC8515648; doi:10.1186/s12913-021-07124-6)
Supplement: Supplementary file 3 — Additional file 3. DU90% of CVDs and diabetes medicines. [file 12913_2021_7124_MOESM3_ESM.docx]

**Additional file 3.** DU90% of CVDs and diabetes medicines.

|  | **Number** | **Medicine (ATC5)** | **DID (n)*** | **DID (%)** |
| --- | --- | --- | --- | --- |
| **CVD medicine** | 1 | Acetylsalicylic acid (B01AC06) | 60.84 | 20.14 |
|  | 2 | Rosuvastatin (C10AA07) | 47.56 | 15.74 |
|  | 3 | Clopidogrel (B01AC04) | 23.63 | 7.82 |
|  | 4 | Atorvastatin (C10AA05) | 22.04 | 7.30 |
|  | 5 | Amlodipine (C08CA01) | 13.09 | 4.33 |
|  | 6 | Ramipril (C09AA05) | 9.76 | 3.23 |
|  | 7 | Valsartan and amlodipine (C09DB01) | 7.33 | 2.43 |
|  | 8 | Losartan and diuretics (C09DA01) | 6.80 | 2.25 |
|  | 9 | Fenofibrate (C10AB05) | 5.91 | 1.96 |
|  | 10 | Bisoprolol (C07AB07) | 5.83 | 1.93 |
|  | 11 | Valsartan and diuretics (C09DA03) | 5.64 | 1.87 |
|  | 12 | Valsartan (C09CA03) | 4.70 | 1.56 |
|  | 13 | Valsartan. amlodipine and hydrochlorothiazide (C09DX01) | 4.52 | 1.50 |
|  | 14 | Atenolol (C07AB03) | 4.49 | 1.49 |
|  | 15 | Isosorbide dinitrate (C01DA08) | 4.24 | 1.40 |
|  | 16 | Pitavastatin (C10AA08) | 4.18 | 1.38 |
|  | 17 | Furosemide (C03CA01) | 4.15 | 1.37 |
|  | 18 | Metoprolol and thiazides (C07BB02) | 4.07 | 1.35 |
|  | 19 | Candesartan (C09CA06) | 4.02 | 1.33 |
|  | 20 | Metoprolol (C07AB02) | 3.30 | 1.09 |
|  | 21 | Platelet aggregation inhibitor combinations (B01AC30) | 3.17 | 1.05 |
|  | 22 | Nebivolol (C07AB12) | 3.14 | 1.04 |
|  | 23 | Atenolol and other diuretics (C07CB03) | 2.98 | 0.99 |
|  | 24 | Bisoprolol and thiazides (C07BB07) | 2.94 | 0.97 |
|  | 25 | Benazepril and amlodipine (C09BB) | 2.93 | 0.97 |
|  | 26 | Candesartan and diuretics (C09DA06) | 2.83 | 0.94 |
|  | 27 | Telmisartan (C09CA07) | 2.44 | 0.81 |
|  | 28 | Ezetimibe (C10AX09) | 2.28 | 0.75 |
|  | 29 | Perindopril (C09AA04) | 2.09 | 0.69 |
|  | 30 | Ramipril and diuretics (C09BA05) | 2.02 | 0.67 |
|  | **Total** | | 272.92 | 90.35 |
| **Diabetes medicines** | 1 | Metformin (A10BA02) | 8.29 | 23.24 |
|  | 2 | Gliclazide (A10BB09) | 7.44 | 20.86 |
|  | 3 | Metformin and sulfonylureas (A10BD02) | 6.82 | 19.13 |
|  | 4 | Metformin and sitagliptin (A10BD07) | 3.06 | 8.59 |
|  | 5 | Glimepiride (A10BB12) | 2.62 | 7.35 |
|  | 6 | Sitagliptin (A10BH01) | 2.34 | 6.57 |
|  | 7 | Pioglitazone (A10BG03) | 1.34 | 3.75 |
|  | 8 | Glipizide (A10BB07) | 0.85 | 2.37 |
|  | **Total** | | 32.76 | 91.86 |

*The drug utilization 90% profile is expressed as the number of defined daily doses per 1,000 people per day (DID).
