# Supplementary material for: Outpatient cardiovascular diseases and diabetes medicines dispensing in the population with government health insurance in Syria between 2018 and 2019: a retrospective analysis
Source: BMC Health Serv Res. 2021 Oct 13;21:1088. doi: 10.1186/s12913-021-07124-6 (PMC8515648; doi:10.1186/s12913-021-07124-6)
Supplement: Supplementary file 4 — Additional file 4. Adjusted insulin dispensing rates (DDED) among adults with health insurance in Syria by governorate. [file 12913_2021_7124_MOESM4_ESM.docx]

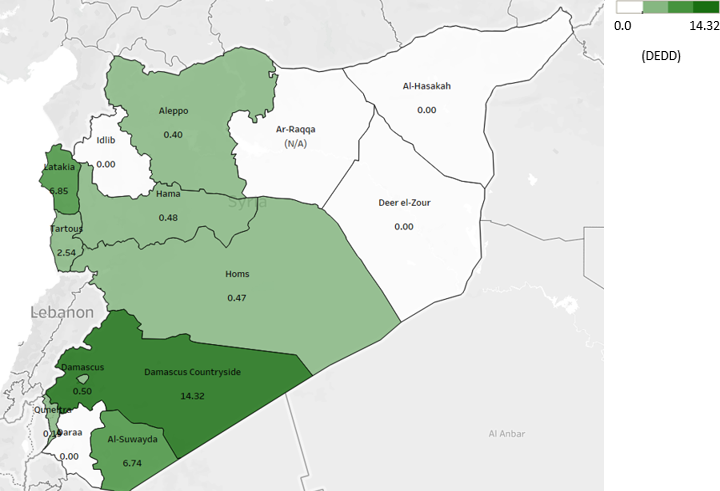


**Additional file 4**. Adjusted insulin dispensing rates (DDED) among adults with health insurance in Syria by governorate.
